# Skin Cancer Detection: A Review Using Deep Learning Techniques

**DOI:** 10.3390/ijerph18105479

**Published:** 2021-05-20

**Authors:** Mehwish Dildar, Shumaila Akram, Muhammad Irfan, Hikmat Ullah Khan, Muhammad Ramzan, Abdur Rehman Mahmood, Soliman Ayed Alsaiari, Abdul Hakeem M Saeed, Mohammed Olaythah Alraddadi, Mater Hussen Mahnashi

**Affiliations:** 1 Government Associate College for Women Mari Sargodha, Sargodha 40100, Pakistan; mehwishdildar94@gmail.com; 2 Department of Computer Science and Information Technology, University of Sargodha, Sargodha 40100, Pakistan; shumailarana_19@yahoo.com; 3 Electrical Engineering Department, College of Engineering, Najran University Saudi Arabia, Najran 61441, Saudi Arabia; miditta@nu.edu.sa; 4 Department of Computer Science, Wah Campus, Comsats University, Wah Cantt 47040, Pakistan; hikmat.ullah@ciitwah.edu.pk; 5 Department of Computer Science, School of Systems and Technology, University of Management and Technology, Lahore 54782, Pakistan; 6 Department of Computer Science, COMSATS University Islamabad, Islamabad 440000, Pakistan; abdurrehman.mahmood@gmail.com; 7 Department of Internal Medicine, Faculty of Medicine, Najran University, Najran 61441, Saudi Arabia; s-alsaiary2@hotmail.com; 8 Department of Dermatology, Najran University Hospital, Najran 61441, Saudi Arabia; hakeeemsaeeed@gmail.com; 9 Department of Internal Medicine, Faculty of Medicine, University of Tabuk, Tabuk 71491, Saudi Arabia; dr.m.o.alraddadi@gmail.com; 10 Department of Medicinal Chemistry, Pharmacy School, Najran University, Najran 61441, Saudi Arabia; matermaha@gmail.com

**Keywords:** deep learning, deep neural network (DNN), machine learning, melanoma, support vector machine (SVM), skin lesion

## Abstract

Skin cancer is one of the most dangerous forms of cancer. Skin cancer is caused by un-repaired deoxyribonucleic acid (DNA) in skin cells, which generate genetic defects or mutations on the skin. Skin cancer tends to gradually spread over other body parts, so it is more curable in initial stages, which is why it is best detected at early stages. The increasing rate of skin cancer cases, high mortality rate, and expensive medical treatment require that its symptoms be diagnosed early. Considering the seriousness of these issues, researchers have developed various early detection techniques for skin cancer. Lesion parameters such as symmetry, color, size, shape, etc. are used to detect skin cancer and to distinguish benign skin cancer from melanoma. This paper presents a detailed systematic review of deep learning techniques for the early detection of skin cancer. Research papers published in well-reputed journals, relevant to the topic of skin cancer diagnosis, were analyzed. Research findings are presented in tools, graphs, tables, techniques, and frameworks for better understanding.

## 1. Introduction

Skin cancer is one of the most active types of cancer in the present decade [1]. As the skin is the body’s largest organ, the point of considering skin cancer as the most common type of cancer among humans is understandable [2]. It is generally classified into two major categories: melanoma and nonmelanoma skin cancer [3]. Melanoma is a hazardous, rare, and deadly type of skin cancer. According to statistics from the American Cancer Society, melanoma skin cancer cases are only 1% of total cases, but they result in a higher death rate [4]. Melanoma develops in cells called melanocytes. It starts when healthy melanocytes begin to grow out of control, creating a cancerous tumor. It can affect any area of the human body. It usually appears on the areas exposed to sun rays, such as on the hands, face, neck, lips, etc. Melanoma type of cancers can only be cured if diagnosed early; otherwise, they spread to other body parts and lead to the victim’s painful death [5]. There as various types of melanoma skin cancer such as nodular melanoma, superficial spreading melanoma, acral lentiginous, and lentigo maligna [3]. The majority of cancer cases lie under the umbrella of nonmelanoma categories, such as basal cell carcinoma (BCC), squamous cell carcinoma (SCC), and sebaceous gland carcinoma (SGC). BCC, SGC, and SCC are formed in the middle and upper layers of the epidermis, respectively. These cancer cells have a low tendency of spreading to other body parts. Nonmelanoma cancers are easily treated as compared with melanoma cancers.

**Figure 1:**
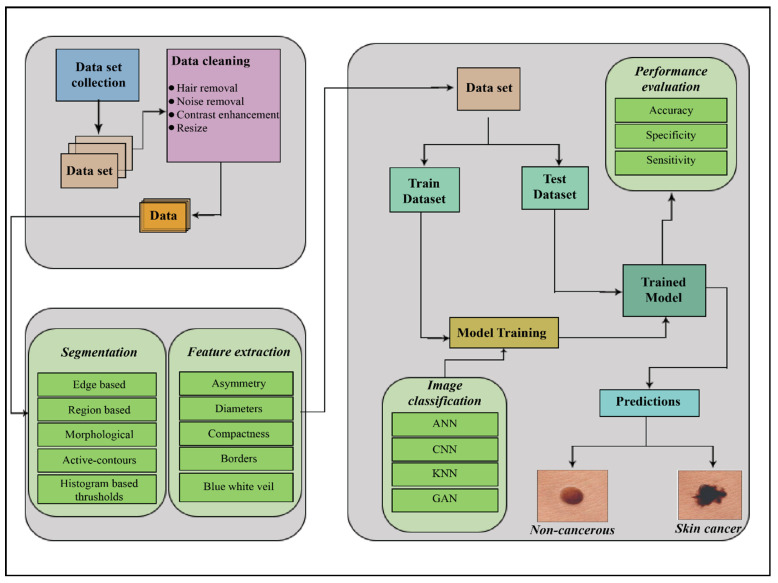
The process of skin cancer detection. ANN = Artificial neural network; CNN = Convolutional neural network; KNN = Kohonen self-organizing neural network; GAN = Generative adversarial neural network.

Therefore, the critical factor in skin cancer treatment is early diagnosis [6]. Doctors ordinarily use the biopsy method for skin cancer detection. This procedure removes a sample from a suspected skin lesion for medical examination to determine whether it is cancerous or not. This process is painful, slow, and time-consuming. Computer-based technology provides a comfortable, less expensive, and speedy diagnosis of skin cancer symptoms. In order to examine the skin cancer symptoms, whether they represent melanoma or nonmelanoma, multiple techniques, noninvasive in nature, are proposed. The general procedure followed in skin cancer detection is acquiring the image, preprocessing, segmenting the acquired preprocessed image, extracting the desired feature, and classifying it, represented in Figure 1.

Deep learning has revolutionized the entire landscape of machine learning during recent decades. It is considered the most sophisticated machine learning subfield concerned with artificial neural network algorithms. These algorithms are inspired by the function and structure of the human brain. Deep learning techniques are implemented in a broad range of areas such as speech recognition [7], pattern recognition [8], and bioinformatics [9]. As compared with other classical approaches of machine learning, deep learning systems have achieved impressive results in these applications. Various deep learning approaches have been used for computer-based skin cancer detection in recent years. In this paper, we thoroughly discuss and analyze skin cancer detection techniques based on deep learning. This paper focuses on the presentation of a comprehensive, systematic literature review of classical approaches of deep learning, such as artificial neural networks (ANN), convolutional neural networks (CNN), Kohonen self-organizing neural networks (KNN), and generative adversarial neural networks (GAN) for skin cancer detection.

A significant amount of research has been performed on this topic. Thus, it is vital to accumulate and analyze the studies, classify them, and summarize the available research findings. To conduct a valuable systematic review of skin cancer detection techniques using deep neural network-based classification, we built search strings to gather relevant information. We kept our search focused on publications of well-reputed journals and conferences. We established multi-stage selection criteria and an assessment procedure, and on the basis of the devised search, 51 relevant research papers were selected. These papers were thoroughly evaluated and analyzed from different aspects. We are greatly encouraged by the trends in skin cancer detection systems, but still, there is space for further improvement in present diagnostic techniques.

This paper is subdivided into four main sections. Section 2 describes the research methodology for performing the effective analysis of deep learning techniques for skin cancer (SC) detection. It contains a description of the review domain, search strings, search criteria, the sources of information, the information extraction framework, and selection criteria. Selected research papers are evaluated, and a detailed survey of SC detection techniques is presented in Section 3. Section 4 summarizes the whole study and presents a brief conclusion.

## 2. Research Methodology

The purpose of performing this systematic literature review was to select and categorize the best available approaches to skin cancer detection using neural networks (NNs). Systematic literature reviews collect and analyze existing studies according to predefined evaluation criteria. Such reviews help to determine what is already known in the concerned domain of study [10].

All data collected from primary sources are organized and analyzed. Once systematic literature is completed, it provides a more sensible, logical, and robust answer to the underlying question of the research [11].

The population of studies considered in the current systematic literature review consisted of research papers relevant to SC detection based on deep neural network (DNN) techniques.

### 2.1. Research Framework

Defining the review framework was the first step in this systematic review. It consisted of an overall plan being followed in the systematic literature review. The plan consisted of three layers: a planning layer, a data selection and evaluation layer, and a results-generation and conclusion layer.

#### 2.1.1. Research Questions

For conducting an effective systematic literature review on a topic, it is necessary to formulate research questions. The research questions formulated for the current systematic research were as follows:

Question No. 1: What are the major deep learning techniques for skin cancer detection?

Question No. 2: What are the main characteristics of datasets available for skin cancer?

#### 2.1.2. Search Strategy

A systematic and well-planned search is very important for collecting useful material from the searched data of the desired domain. In this step, a thorough search was conducted to extract meaningful and relevant information from the mass of data. We created an automated search mechanism for filtering out the desired domain’s data from all sources. Research papers, case studies, American Cancer Society reports, and reference lists of related publications were examined in detail. Websites containing information regarding skin cancer, the dangers of skin cancer, the reasons for skin cancer, and NN techniques of skin cancer detection were all carefully searched. For extraction of the desired and relevant data, we conducted our search according to the following parameters.

- Search keywords/search term identification based on research questions
- Words related to the search keywords
- Search string formulation using logical operators between search words

The keywords related to deep learning techniques for skin cancer detection were selected. Subsequently, the search was extended to synonyms for these keywords.

Furthermore, the search was carried out using logical operators ’AND’ and ‘OR’ between keywords. The keywords used to search information relevant to skin cancer are listed in Table 1.

#### 2.1.3. Resources of Search

We conducted our initial search on well-reputed search engines such as IEEE Xplore, ACM, Springer as well as Google Scholar to extract information relevant to NN techniques for skin cancer detection. Basic research material related to the underlying topic was filtered out in the primary search. The selected research papers and conference proceedings were further analyzed according to evaluation criteria.

#### 2.1.4. Initial Selection Criteria

The initial selection of research papers/conference papers was based on certain specified parameters such as the language of the paper, the year of the paper, and the relevance of the topic within the desired domain. Only research papers written in the English language were included in this research. Our review paper focused on research published between 2011 and 2021. Selected papers had to be relevant to the search terms described in the search strategy.

### 2.2. Selection and Evaluation Procedure

Using the initial search criteria, the search extracted 1483 research papers and conference reports. From the papers that were identified, we selected 95 papers that had a title considered relevant to our study. Subsequently, the abstracts of those selected papers were examined more closely for their relevance, which led to reducing their number to 64 research papers. The research papers successfully passing abstract-based selection were studied in detail. The quality of those research papers was fully examined, and 51 research papers were selected for final review. In this finalized selection, the percentage of IEEE publications was 25%, Google Scholar’s selection percentage was 16%, 10% papers were selected from ACM DL, 29% from springer, and 20% from Science Direct. The search results are represented in Table 2.

A thorough study of the full text of the selected research papers sought answers to certain quality control questions. The current systematic research asked the following quality assessment questions.

- Did the selected study cover all aspects of this review’s topic?
- Was the quality of the selected paper verified?
- Does the selected study adequately answer the research questions?

The first quality assessment question focused on the thorough coverage of deep learning techniques for skin cancer detection. The quality of a selected paper was verified by the reputation of the journal in which it was published and by its citations. The third question ensured that the research answered the research questions mentioned in Section 2. Only the most relevant research papers to our domain of study were extracted. These papers had to satisfy those above research questions to qualify for selection. Research papers that failed to adequately answer the research or quality control questions and papers with text that was not related to our study topic were excluded.

Each question had Boolean ’yes/no’ responses. Each ‘yes’ was assigned a value Y = 1 and each ‘no’ was assigned a value N = 0. The first quality control question evaluated the topic coverage of the 51 selected research papers and resulted in a value of 77%, which was quite satisfactory. The second research question verified the quality of the selected papers, which resulted in improvement of quality. It generated an 82% result, which was satisfactory. The third question was a very important question in order to answer the review’s main research questions. It generated a 79% result, which was the indicator of the adequacy of the studies to answer the research questions posed by the review. The overall results of answers to these quality questions seemed healthy.

## 3. Deep Learning Techniques for Skin Cancer Detection

**Figure 2:**
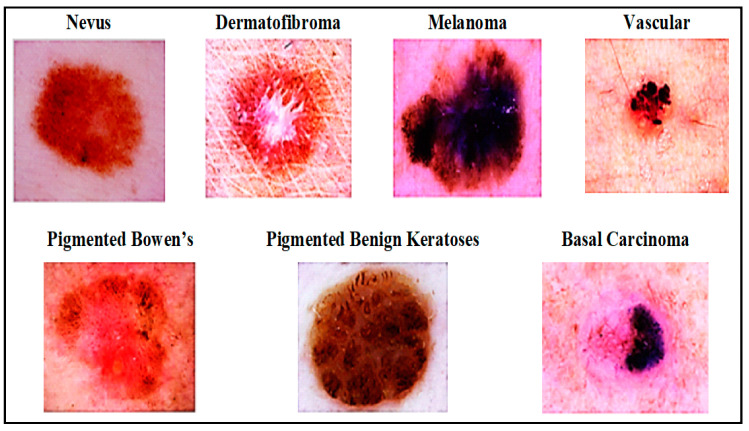
Skin disease categories from International Skin Imaging Collaboration (ISIC) dataset [12].

Deep neural networks play a significant role in skin cancer detection. They consist of a set of interconnected nodes. Their structure is similar to the human brain in terms of neuronal interconnectedness. Their nodes work cooperatively to solve particular problems. Neural networks are trained for certain tasks; subsequently, the networks work as experts in the domains in which they were trained. In our study, neural networks were trained to classify images and to distinguish between various types of skin cancer. Different types of skin lesion from International Skin Imaging Collaboration (ISIC) dataset are presented in Figure 2. We searched for different techniques of learning, such as ANN, CNN, KNN, and GAN for skin cancer detection systems. Research related to each of these deep neural networks is discussed in detail in this section.

### 3.1. Artificial Neural Network (ANN)-Based Skin Cancer Detection Techniques

**Figure 3:**
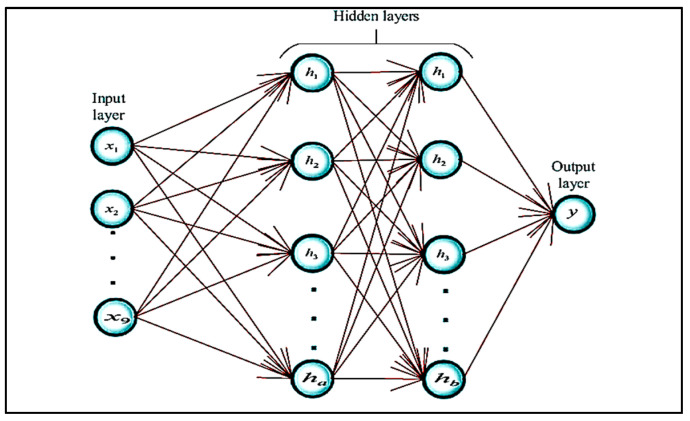
Basic ANN structure [13].

An artificial neural network is a nonlinear and statistical prediction method. Its structure is borrowed from the biological structure of the human brain. An ANN consists of three layers of neurons. The first layer is known as the input layer; these input neurons transfer data to the second/intermediate layer of neurons. The intermediate layers are referred to as hidden layers. In a typical ANN, there can be several hidden layers. Intermediate neurons send data to the third layer of output neurons. Computations are learned at each layer using backpropagation, which is used for learning the complex associations/relationships between input and output layers. It is similar to a neural network. Currently, in computer science, the term neural network and artificial neural network are used interchangeably. The basic structure of an ANN network is presented in Figure 3.

ANN is used for the classification of extracted features in skin cancer detection systems. Input images are classified as melanoma or nonmelanoma after successful training/classification of the training set. The number of hidden layers in an ANN depends on the number of input images. The input/first layer of the ANN process connects with the hidden layer by the input dataset. The dataset can be labeled or unlabeled, which can be processed accordingly using a supervised or unsupervised learning mechanism. A neural network uses backpropagation or feed-forward architecture to learn weights present at each network connection/link. Both architectures use a different pattern for the underlying dataset. Feed-forward-architecture-based neural networks transfer data only in one direction. Data flows only from the input to the output layer.

Xie et al. [14] proposed a skin lesion classification system that classified lesions into two main classes: benign and malignant. The proposed system worked in three phases. In the initial phase, a self-generating NN was used to extract lesions from images. In the second phase, features such as tumor border, texture, and color details were extracted. The system extracted a total of 57 features, including 7 novel features related to lesion borders descriptions. Principal component analysis (PCA) was used to reduce the dimensionality of the features, which led to the selection of the optimal set of features. Finally, in the last phase, lesions were classified using a NN ensemble model. Ensemble NN improves classification performance by combining backpropagation (BP) NN and fuzzy neural networks. Furthermore, the proposed system classification results were compared with other classifiers, such as SVM, KNN, random forest, Adaboot, etc. With a 91.11% accuracy, the proposed model achieved at least 7.5% higher performance in terms of sensitivity than the other classifiers.

Masood et al. [15] proposed an ANN-based automated skin cancer diagnostic system. The performance of three ANN’s learning algorithms such as Levenberg–Marquardt (LM) [16], resilient backpropagation (RP) [17], scaled conjugate gradient (SCG) [18], was also investigated by this paper. Comparison of performance showed that the LM algorithm achieved the highest specificity score (95.1%) and remained efficient at the classification of benign lesions, while the SCG learning algorithm produced better results if the number of epochs was increased, scoring a 92.6% sensitivity value. A mole classification system for the early diagnosis of melanoma skin cancer was proposed [19]. The proposed system extracted features according to the ABCD rule of lesions. ABCD refers to asymmetry of a mole’s form, borders of mole, color, and diameter of mole. Assessment of a mole’s asymmetry and borders were extracted using the Mumford–Shah algorithm and Harris Stephen algorithm, respectively. Normal moles are composed of black, cinnamon, or brown color, so moles with colors other than those three were considered melanoma in the proposed system. Melanoma moles commonly have a diameter value greater than 6 mm, so that value was used as the threshold value of diameter for melanoma detection. The proposed system used a backpropagation feed-forward ANN to classify moles into three classes, such as common mole, uncommon mole, or melanoma mole, with 97.51% accuracy.

**Figure 4:**
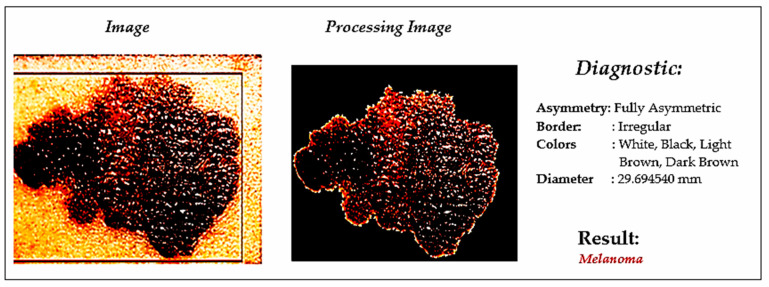
Skin cancer detection using ANN [19].

An automated skin cancer diagnostic system based on backpropagation ANN was proposed [20], represented in Figure 4. This system employed a 2D-wavelet transform technique for feature extraction. The proposed ANN model classified the input images into two classes, such as cancerous or noncancerous. Another ANN-based skin cancer diagnostic system was proposed by Choudhari and Biday [21]. Images were segmented with a maximum entropy thresholding measure. A gray-level co-occurrence matrix (GLCM) was used to extract unique features of skin lesions. Finally, a feed-forward ANN classified the input images into either a malignant or benign stage of skin cancer, achieving an accuracy level of 86.66%.

Aswin et al. [22] described a new method for skin cancer detection based on a genetic algorithm (GA) and ANN algorithms. Images were preprocessed for hair removal with medical imaging software named Dull-Rozar and region of interest (ROI) and were extracted with the Otsu thresholding method. Furthermore, the GLCM technique was employed to extract unique features of the segmented images. Subsequently, a hybrid ANN and GA classifier was used for the classification of lesion images into cancerous and noncancerous classes. The proposed system achieved an overall accuracy score of 88%. Comprehensive details of the various skin cancer detection systems based on ANN are listed in Table 3 below.

### 3.2. Convolutional Neural Network (CNN)-Based Skin Cancer Detection Techniques

**Figure 5:**
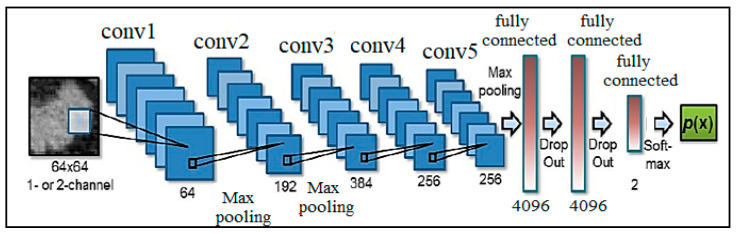
Basic CNN Architecture [9].

A convolution neural network is an essential type of deep neural network, which is effectively being used in computer vision. It is used for classifying images, assembling a group of input images, and performing image recognition. CNN is a fantastic tool for collecting and learning global data as well as local data by gathering more straightforward features such as curves and edges to produce complex features such as shapes and corners [28]. CNN’s hidden layers consist of convolution layers, nonlinear pooling layers, and fully connected layers [29]. CNN can contain multiple convolution layers that are followed by several fully connected layers. Three major types of layers involved in making CNN are convolution layers, pooling layers, and full-connected layers [30]. The basic architecture of a CNN is presented in Figure 5.

CNN-based automated deep learning algorithms have achieved remarkable performance in the detection, segmentation, and classification operations of medical imaging [31]. Lequan et al. [32] proposed a very deep CNN for melanoma detection. A fully convolutional residual network (FCRN) having 16 residual blocks was used in the segmentation process to improve performance. The proposed technique used an average of both SVM and softmax classifier for classification. It showed 85.5% accuracy in melanoma classification with segmentation and 82.8% without segmentation. DeVries and Ramachandram [33] proposed a multi-scale CNN using an inception v3 deep neural network that was trained on an ImageNet dataset. For skin cancer classification, the pre-trained inception v3 was further fined-tuned on two resolution scales of input lesion images: coarse-scale and finer scale. The coarse-scale was used to capture shape characteristics as well as overall contextual information of lesions. In contrast, the finer scale gathered textual detail of lesion for differentiation between various types of skin lesions.

Mahbod et al. [34] proposed a technique to extract deep features from various well-established and pre-trained deep CNNs for skin lesions classification. Pretrained AlexNet, ResNet-18 and VGG16 were used as deep-feature generators, then a multi-class SVM classifier was trained on these generated features. Finally, the classifier results were fused to perform classification. The proposed system was evaluated on the ISIC 2017 dataset and showed 97.55% and 83.83% area under the curve (AUC) performance for seborrheic keratosis (SK) and melanoma classification. A deep CNN architecture based on pre-trained ResNet-152 was proposed to classify 12 different kinds of skin lesions [35]. Initially, it was trained on 3797 lesion images; however, later, 29-times augmentation was applied based on lighting positions and scale transformations. The proposed technique provided an AUC value of 0.99 for the classification of hemangioma lesion, pyogenic granuloma (PG) lesion, and intraepithelial carcinoma (IC) skin lesions.

**Figure 6:**
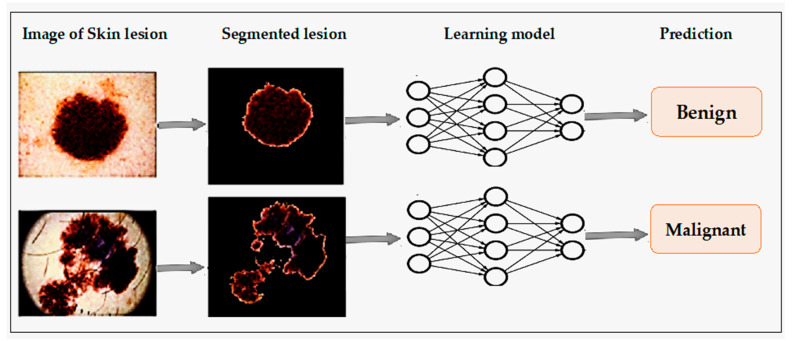
Skin cancer diagnosis using CNN [37].

A technique for the classification of four different types of skin lesion images was proposed by Dorj et al. [36]. A pre-trained deep CNN named AlexNet was used for feature extraction, after which error-correcting output coding SVM worked as a classifier. The proposed system produced the highest scores of the average sensitivity, specificity, and accuracy for SCC, actinic keratosis (AK), and BCC: 95.1%, 98.9%, and 94.17%, respectively. Kalouche [37] proposed a pre-trained deep CNN architecture VGG-16 with a final three fine-tuned layers and five convolutional blocks. The proposed VCG-16 model is represented in Figure 6. VCG-16 models showed 78% accuracy for the classification of lesion images as melanoma skin cancer. A deep CNN-based system was proposed to detect the borders of skin lesions in images. The deep learning model was trained on 1200 normal skin images and 400 images of skin lesions. The proposed system classified the input images into two main classes, normal skin image and lesion image, with 86.67% accuracy. A comprehensive list of skin cancer detection systems using CNN classifiers is presented in Table 4.

### 3.3. Kohonen Self-Organizing Neural Network (KNN)-Based Skin Cancer Detection Techniques

The Kohonen self-organizing map is a very famous type of deep neural network. CNNs are trained on the basis of unsupervised learning, which means that a KNN does not require any developer’s intervention in the learning process as well as requiring little information about the attributes of the input data. A KNN generally consists of two layers. In the 2-D plane, the first layer is called an input layer, while another is named a competitive layer. Both of these layers are fully connected, and every connection is from the first to second layer dimension. A KNN can be used for data clustering without knowing the relationships between input data members. It is also known as a self-organizing map. KNNs do not contain an output layer; every node in the competitive layer also acts as the output node itself.

**Figure 7:**
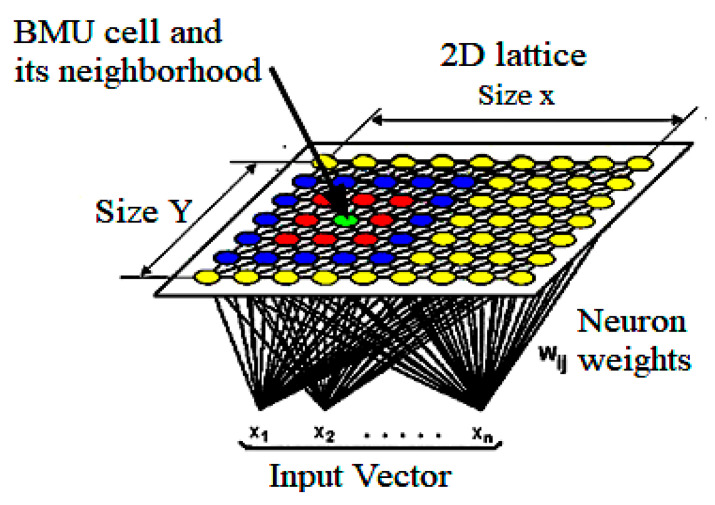
Basic KNN structure [58], BMU= Best matching unit.

A KNN basically works as a dimensionality reducer. It can reduce the high dimensional data into a low dimension, such as a two-dimensional plane. Thus, it provides discrete types of representation of the input dataset. KNNs are different from other types of NN in terms of learning strategy because it uses competitive learning rather than the learning based on error correction found in BPN or feed-forward learning. A KNN preserves the topological structure of the input data space during mapping dimensionality from high to low. Preservation refers to the preservation of relative distance between data points in space. Data points that are closer in input data space are mapped closer to each other in this scheme; far points are mapped far from each other as well as, according to the relative distance present among them. Consequently, a KNN is the best tool for high dimensional data. Another important feature provided by a KNN is its generalization ability. The network has the ability to recognize and organize unknown input data. The architecture of a KNN is shown in Figure 7. A KKN’s main quality is its ability to map complex relationships of data points in which even nonlinear relations exist between data points. Due to these benefits, nowadays, KNNs are being used in skin cancer detection systems.

Lenhardt et al. [59] proposed a KNN-based skin cancer detection system. The proposed system processed synchronous fluorescence spectra of melanoma, nevus, and normal skin samples for neural network training. A fluorescence spectrophotometer was used to measure the fluorescence spectra of the samples, whereas samples were collected from human patients immediately after surgical resection. The dimensionality of measured spectra was reduced with the PCA technique. Both KNN and ANN were trained, and their performance for melanoma detection was compared. On the test dataset, the classification error of KNN was 2–3%, while the classification error for ANN lay in the range of 3% to 4%.

A combination of self-organizing NN and radial basis function (RBF) neural network was proposed to diagnose three different types of skin cancer, such as BCC, melanoma, and SCC [60]. The proposed system extracted color, GLCM, and morphological features of lesion images, after which the classification model used those features as input. Furthermore, the classification performance of the proposed system was compared with *k*-nearest neighbor, ANN, and naïve-Bayes classifiers. The proposed system achieved 93.150685% accuracy while *k*-nearest neighbor showed 71.232877%, ANN showed 63.013699%, and naïve Bayes showed 56.164384% accuracy scores.

Another KNN-based automated skin cancer diagnostic system was proposed by Sajid et al. [61]. The proposed system employed a median filter as a noise removal technique. Then filtered images were segmented with a statistical region growing and merging technique. In this system, a collection of textual and statistical features was used. Statistical features were extracted from lesion images, whereas textual features were extracted from a curvelet domain. Finally, the proposed system classified the input images into cancerous or noncancerous with 98.3% accuracy. In this work, other classifiers such as SVM, BPN, and 3-layer NN were also implemented, and their performance was compared with the proposed system’s classification performance. SVM produced 91.1% accuracy, BPN showed 90.4% accuracy, 3-layer NN showed 90.5%, whereas the proposed system achieved the highest accuracy of 98.3% for skin cancer diagnosis. Details on the KNN-based skin cancer diagnostic systems is presented in Table 5.

### 3.4. Generative Adversarial Network (GAN)-Based Skin Cancer Detection Techniques

**Figure 8:**
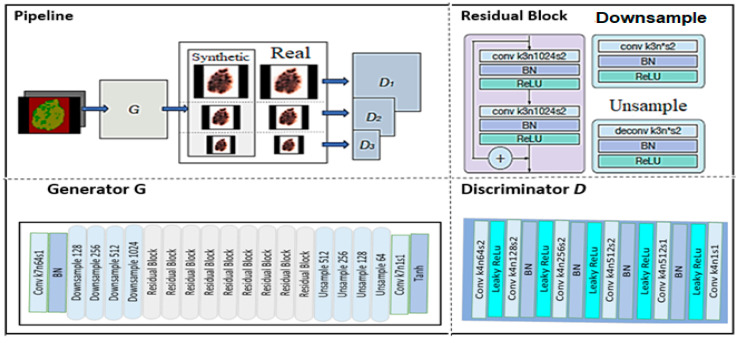
GAN architecture [64].

A generative adversarial neural network is a powerful class of DNN that is inspired by zero-sum game theory [62]. GANs are based on the idea that two neural networks, such as a generator and a discriminator, compete with each other to analyze and capture the variance in a database. The generator module uses the data distribution to produce fake data samples and tries to misguide the discriminator module. On the other hand, the discriminator module aims to distinguish between real and fake data samples [63]. In the training phase, both of these neural networks repeat these steps, and their performance improves after each competition. The ability to generate fake samples that are similar to a real sample using the same data distribution, such as photorealistic images, is the major power of a GAN network. It can also solve a major problem in deep learning: the insufficient training examples problem. Research scholars have been implementing various types of GANs, such as Vanilla GAN, condition GAN (CGAN), deep convolutional GAN (DCGAN), super-resolution GAN (SRGAN), and Laplacian Pyramid GAN (LPGAN). Nowadays, GANs are successfully being used in skin cancer diagnostic systems. The architecture of a GAN is shown in Figure 8.

Rashid et al. [7] proposed a GAN-based skin lesion classification system. The proposed system performed augmentation on a training set of images with realistic-looking skin lesion images generated via GAN. A deconvolutional network was used as the generator module, while the discriminator module used CNN as a classifier. The CNN learned to classify seven different categories of skin lesions. Results of the proposed system were compared with ResNet-50 and DenseNet. ResNet-50 produced 79.2% accuracy, DenseNet showed 81.5% accuracy, whereas the proposed approach achieved the highest accuracy of 86.1% for skin lesion classification. Deep learning methods provide sufficient accuracy but require pure, unbalanced, and large training datasets. To overcome these limitations, Bisla et al. [8] proposed a deep learning approach for data purification and GAN for data augmentation. The proposed system used decoupled deep convolutional GANs for data generation. A pre-trained ResNet-50 model was further refined with a purified and augmented dataset and was used to classify dermoscopic images into three categories: melanoma, SK, and nevus. The proposed system outperformed the baseline ResNet-50 model for skin lesion classification and achieved 86.1% accuracy.

A novel data augmentation method for a skin lesion on the basis of self-attention progressive GAN (PGAN) was proposed. Moreover, the generative model was enhanced with the stabilization technique. The proposed system achieved 70.1% accuracy as compared with 67.3% accuracy produced by a non-augmented system. A list of GAN-based skin cancer detection systems with their diagnosed skin cancer type, classifier, dataset, and the obtained result is presented in Table 6.

## 4. Datasets

Several computer-based systems for skin cancer diagnosis have been proposed. Evaluating their diagnostic performance and validating predicted results requires a solid and reliable collection of dermoscopic images. Various skin cancer datasets have lacked size and diversity other than for images of nevi or melanoma lesions. Training of artificial neural networks for skin lesion classification is hampered by the small size of the datasets and a lack of diverse data. Although patients commonly suffer from a variety of non-melanocytic lesions, past research for automated skin cancer diagnosis primarily focused on diagnosing melanocytic lesions, resulting in a limited number of diagnoses in the available datasets [66]. Therefore, the availability of a standard, reliable dataset of dermoscopic images is very crucial. Real-world datasets for the evaluation of proposed skin cancer detection techniques are discussed in this section. Table 7 summarizes the important details of these datasets.

### 4.1. HAM10000

There is a human-against-machine dataset with 10,000 training images that is referred to as HAM10000 [66]. It is the latest publicly available skin lesions dataset, and it overcomes the problem of the lack of diversity. The final dataset of HAM10000 contains 10,015 dermoscopic images, collected from two sources: Cliff Rosendahl’s skin cancer practice in Queensland, Australia, and the Dermatology Department of the Medical University of Vienna, Austria. This collection has taken twenty years to compile. Before widespread use of digital cameras, photographic prints of lesions were deposited and stored at the Dermatology Department of the Medical University of Vienna, Austria. These photographic prints were digitalized with the help of Nikon-Coolscan-5000-ED scanner, manufactured by Nikon corporation Japan and converted into 8-bit color JPEG images having 300 DPI quality. The images were then manually cropped and saved at 800 × 600 pixels resolution at 72 DPI.

Several acquisition functions and cleaning methods were applied to the images and a semi-automatic workflow was developed using a neural network to attain diversity. The resulting dataset contains 327 images of AK, 514 images of basal cell carcinomas, 1099 images of benign keratoses, 115 images of dermatofibromas, 1113 images of melanocytic nevi, 6705 images of melanomas, and 142 images of vascular skin lesions.

### 4.2. PH2

The dermoscopic images in the PH² dataset were collected at the Dermatology Center of Pedro Hispano Hospital, Portugal [68]. These images were obtained using a Tuebinger-Mole-Analyzer system under the same conditions and magnification rate of 20×. PH2 dataset contains 8-bit RGB color images having 768 × 560 pixels resolution. The dataset contains 200 dermoscopic images, divided into 80 images of common nevi, 80 images of atypical nevi, and 40 images of melanoma skin cancers. This dataset contains medical annotation of the lesion images, such as medical segmentation of pigmented skin lesions, histological and clinical diagnosis, and evaluation of various dermoscopic criteria. The assessment was performed according to dermoscopic criteria of streaks, colors, regression areas, pigment network, and blue-whitish veil globules.

### 4.3. ISIC Archive

The ISIC archive [69] is a collection of various skin lesions datasets. The ISIC dataset [70] was originally released by the International Skin Imaging Collaboration at the International Symposium on Biomedical Imaging (ISBI) challenge 2016, named as ISIC2016. The ISIC2016 archive is divided into two parts: training and testing. The training subset of ISIC contains 900 images, while the testing subset contains 379 dermoscopic images. It includes images of two classes: malignant melanomas and benign nevi. Approximately 30.3% of the dataset’s images are of melanoma lesions and the remaining images belong to the benign nevi class. ISIC increases the number of images in its archive every year and has established a design challenge for the development of a system for skin cancer automated diagnosis.

In the ISIC2017 dataset, there were three categories of images: melanomas, seborrheic-keratoses (SK), and benign nevi. The dataset contains 2000 training images, 150 validation images, and 600 images for testing. The training dataset contains 374 images of melanomas, 254 SK images, and 1372 images of benign nevi. The validation dataset contains 30 melanoma images, 42 SK images, and 78 benign nevus images. The test dataset includes 117 melanoma images, 90 SK images, and 393 benign nevus images. ISIC2018 contains 12,594 training images, 100 validation images, and 1000 test images. The ISIC2019 dataset includes 25,331 images of eight different categories of skin lesions, such as melanoma, melanocytic-nevus, BCC, AK, benign keratosis, dermatofibroma, vascular lesion, and SCC. It contains 8239 images in the test dataset and an additional outlier class that was not included in the training dataset. The new proposed skin cancer diagnostic systems must be able to identify these images. The ISIC2019 dataset also includes metadata for images, such as sex, age, and area of the patient.

### 4.4. Derm Quest

The publicly available DermQuest dataset [71] contained 22,082 dermoscopic images. Among all dermoscopic datasets, only the DermQuest dataset contained lesion tags for skin lesions. There were 134 lesion tags for all images in the dataset. The DermQuest dataset redirected to Derm101 in 2018. However, this dataset was deactivated recently on 31 December 2019.

### 4.5. DermIS

The Dermoscopic dataset Dermatology Information System is commonly known as DermIS [72]. This dataset was built through cooperation between the Department of Dermatology of the University of Erlangen and the Department of Clinical Social Medicine of the University of Heidelberg. It contains 6588 images. This dataset has recently been divided into two parts: a dermatology online image atlas (DOIA) and a pediatric dermatology online image atlas (PeDOIA). The DOIA includes 3000 lesion images covering approximately 600 dermatological diagnoses. It provides dermoscopic images complete with differential and provisional diagnoses, case reports, and other information on nearly all types of skin diseases.

### 4.6. AtlasDerm

The Atlas of Dermoscopy dataset is commonly referred to as AtlasDerm [73]. It is a unique and well-organized combination of a book and images on CD-ROM with sample examples for training. It was originally designed as a tool to help physicians in the diagnosis of skin lesions and the recognition of dermoscopic criteria related to melanoma. The AtlasDerm dataset considers various cases of skin lesions, with corresponding dermoscopic images for every case. It contains 5 images of AK, 42 images of BCC, 70 images of benign keratosis, 20 images of dermatofibroma, 275 images of melanocytic nevus, 582 images of melanoma, and 30 images of vascular skin lesions.

### 4.7. Dermnet

The Dermnet Skin Disease Atlas dataset is commonly referred to as Dermnet [74]. It was built in 1998 by Dr. Thomas Habif in Portsmouth, New Hampshire. It consists of more than 23,000 dermoscopic images. This database contains images of 643 different types of skin diseases. These diseases are biologically organized into a two-level taxonomy. The bottom level contains more than 600 skin diseases in fine granularity. The top-level taxonomy contains 23 different classes of skin diseases, such as connective tissue disease, benign tumors, eczema, melanomas, moles, nevi, etc.

## 5. Open Research Challenges

### 5.1. Extensive Training

One of the major challenges in neural network-based skin cancer detection techniques is the extensive training that is required. In other words, to successfully analyze and interpret the features from dermoscopic images, the system must undergo detailed training, which is a time-consuming process and demands extremely powerful hardware.

### 5.2. Variation in Lesion Sizes

Another challenge is the variation in the sizes of lesions. A group of Italian and Austrian researchers collected many benign and cancerous melanoma lesion images in the 1990s [73]. The diagnostic accuracy of the identification of the lesions was as high as 95% to 96% [75]. However, the diagnostic process, with earlier stage and smaller lesions of 1mm or 2mm in size, was much more difficult and error-prone.

### 5.3. Images of Light Skinned People in Standard Datasets

Existing standard dermoscopic datasets contain images of light-skinned people, mostly from Europe, Australia, and the United States. For accurate skin cancer detection in dark-skinned people, a neural network must learn to account for skin color [76]. However, doing so is possible only if the neural network observes enough images of dark-skinned people during the process of training. Therefore, datasets having sufficient lesion images of dark-skinned and light-skinned people is necessary for increasing the accuracy of skin cancer detection systems.

### 5.4. Small Interclass Variation in Skin Cancer Images

Unlike the other types of images, medical images have very small interclass variation; that is, the difference between melanoma and nonmelanoma skin cancer lesion images has much less variation than, say, the variation between images of cats and dogs. It is also very difficult to differentiate between a birthmark and a melanoma. The lesions of some disease are so similar that it is extremely hard to distinguish them. This limited variation makes the task of image analysis and classification very complex [32].

### 5.5. Unbalanced Skin Cancer Datasets

Real-world datasets used for skin cancer diagnosis are highly unbalanced. Unbalanced datasets contain a very different number of images for each type of skin cancer. For example, they contain hundreds of images of common skin cancer types but only a few images for the uncommon types, making it difficult to draw generalizations from the visual features of the dermoscopic images [12].

### 5.6. Lack of Availability of Powerful Hardware

Powerful hardware resources with high graphical processing unit (GPU) power are required for the NN software to be able to extract the unique features of a lesion’s image, which is critical for achieving better skin cancer detection. The lack of availability of high computing power is a major challenge in deep learning-based skin cancer detection training.

### 5.7. Lack of Availability of Age-Wise Division of Images In Standard Datasets

Various types of skin cancers such as Merkel cell cancer, BCC, and SCC typically appear after the age of 65 years [77]. Existing standard dermoscopic datasets contain images of young people. However, for an accurate diagnosis of skin cancer in elderly patients, it is necessary that neural networks observe enough images of people aged more than 50 years.

### 5.8. Use of Various Optimization Techniques

Preprocessing and detection of lesion edges are very crucial steps in the automated detection of skin cancer. Various optimization algorithms such as artificial the bee colony algorithm [78], ant colony optimization [79], social spider optimization [80], and particle swarm optimization [81] can be explored to increase the performance of automated skin cancer diagnostic systems.

### 5.9. Analysis of Genetic and Environmental Factors

Researchers have identified various genetic risk factors for melanoma, such as fair skin, light colored eyes, red hair, a large number of moles on the body, and a family history of skin cancer. When these genetic risk factors are combined with environmental risks such as high ultraviolet light exposure, the chances of developing skin cancer become very high [82]. These factors can be combined with existing deep learning approaches for better performance.

## 6. Conclusion and Future Work

This systematic review paper has discussed various neural network techniques for skin cancer detection and classification. All of these techniques are noninvasive. Skin cancer detection requires multiple stages, such as preprocessing and image segmentation, followed by feature extraction and classification. This review focused on ANNs, CNNs, KNNs, and RBFNs for classification of lesion images. Each algorithm has its advantages and disadvantages. Proper selection of the classification technique is the core point for best results. However, CNN gives better results than other types of a neural networks when classifying image data because it is more closely related to computer vision than others.

Most of the research related to skin cancer detection focuses on whether a given lesion image is cancerous. However, when a patient asks if a particular skin cancer symptom appears on any part of their body, the current research cannot provide an answer. Thus far, the research has focused on the narrow problem of classification of the signal image. Future research can include full-body photography to seek the answer to the question that typically arises. Autonomous full-body photography will automate and speed up the image acquisition phase.

The idea of auto-organization has recently emerged within the area of deep learning. Auto-organization refers to the process of unsupervised learning, which aims to identify features and to discover relations or patterns in the image samples of the dataset. Under the umbrella of convolutional neural networks, auto-organization techniques increase the level of features representation that is retrieved by expert systems [47]. Currently, auto-organization is a model that is still in research and development. However, its study can improve the accuracy of image processing systems in the future, particularly in the area of medical imaging, where the smallest details of features are extremely crucial for the correct diagnosis of disease.

## Figures and Tables

**Table 1:** Search terms.

Search Term	Set of Keywords
Skin *	Skin cancer, skin diseases, skin treatment
Cancer *	Cancer disease, cancer types, cancer diagnosis, cancer treatment
Deep *	Deep learning, deep neural networks
Neural *	Neural network, neural networking
Network *	Neural network, neural networking
Melano *	Networking, network types
NonMelano *	Melanoma skin cancer, melanoma death rate, melanoma treatment, melanoma diagnosis, melanoma causes, melanoma symptoms
Basal *	Basal cell carcinoma, basal cell carcinoma skin cancer, basal cell carcinoma diagnosis, basal cell carcinoma causes, basal cell carcinoma symptoms
Squamous *	Squamous cell carcinoma, squamous cell carcinoma skin cancer, squamous cell carcinoma diagnosis, squamous cell carcinoma causes, squamous cell carcinoma symptoms
Artificial *	Artificial neural network, artificial neural networking,
Back *	Backpropagation neural network
Conv *	Convolutional neural network

* = All words that start with the string written before asterisk *.

**Table 2:** Search results.

Sr. No	Resource	Initial Search	Title-Based Selection	Abstract-Based Selection	Full Paper-Based Selection
1	IEEE Xplore	123	21	15	13
2	Google Scholar	451	29	11	8
3	ACM DL	327	19	9	5
4	Springer	235	11	17	15
5	Science Direct	347	15	12	10
	Total	1483	95	64	51

**Table 3:** A comparative analysis of skin cancer detection using ANN-based approaches.

Ref	Skin Cancer Diagnoses	Classifier and Training Algorithm	Dataset	Description	Results (%)
[23]	Melanoma	ANN with backpropagation algorithm	31 dermoscopic images	ABCD parameters for feature extraction,	Accuracy (96.9)
[20]	Melanoma/Non- melanoma	ANN with backpropagation algorithm	90 dermoscopic images	maximum entropy for thresholding, and gray- level co-occurrence matrix for features extraction	Accuracy (86.66)
[19]	Cancerous/non- cancerous	ANN with backpropagation algorithm	31 dermoscopic images	2D-wavelet transform for feature extraction and thresholding for segmentation	Nil
[24]	Malignant /benign	Feed-forward ANN with the backpropagation training algorithm	326 lesion images	Color and shape characteristics of the tumor were used as discriminant features for classification	Accuracy (80)
[25]	Malignant/non-Malignant	Backpropagation neural network as NN classifier	448 mixed-type images	ROI and SRM for segmentation	Accuracy (70.4)
[21]	Cancerous/noncancerous	ANN with backpropagation algorithm	30 cancerous/noncancerous images	RGB color features and GLCM techniques for feature extraction	Accuracy (86.66)
[18]	Common mole/non-common mole/melanoma	Feed-forward BPNN	200 dermoscopic images	Features extracted according to ABCD rule	Accuracy (97.51)
[26]	Cancerous/noncancerous	Artificial neural network with backpropagation algorithm	50 dermoscopic images	GLCM technique for feature extraction	Accuracy (88)
[27]	BCC/non-BCC	ANN	180 skin lesion images	Histogram equalization for contrast enhancement	Reliability (93.33)
[14]	Melanoma/Non-melanoma	ANN with Levenberg–Marquardt (LM), resilient backpropagation (RBP), and scaled conjugate gradient (GCG) learning algorithms	135 lesion images	Combination of multiple classifiers to avoid the misclassification	Accuracy (SCG:91.9, LM: 95.1, RBP:88.1)
[13]	Malignant/benign	ANN meta-ensemble model consisting of BPN and fuzzy neural network	Caucasian race and xanthous-race datasets	Self-generating neural network was used for lesion extraction	Accuracy (94.17) Sensitivity (95), specificity (93.75)

ANN = Artificial neural network, NN = Neural network. ROI = Region of interest, SRM = Statistical region merging, GLCM = Gray level co-occurrence matrix, BPNN = Backpropagation neural network.

**Table 4:** A comparative analysis of skin cancer detection using CNN-based approaches.

Ref	Skin Cancer Diagnoses	Classifier and Training Algorithm	Dataset	Description	Results (%)
[38]	Benign/malignant	LightNet (deep learning framework), used for classification	ISIC 2016 dataset	Fewer parameters and well suited for mobile applications	Accuracy (81.6), sensitivity (14.9), specificity (98)
[31]	Melanoma/benign	CNN classifier	170 skin lesion images	Two convolving layers in CNN	Accuracy (81), sensitivity (81), specificity (80)
[36]	BCC/SCC/melanoma/AK	SVM with deep CNN	3753 dermoscopic images	Pertained to deep CNN and AlexNet for features extraction	Accuracy (SCC: 95.1, AK: 98.9, BCC: 94.17)
[39]	Melanoma /benign Keratinocyte carcinomas/benign SK	Deep CNN	ISIC-Dermoscopic Archive	Expert-level performance against 21 certified dermatologists	Accuracy (72.1)
[35]	Malignant melanoma and BC carcinoma	CNN with Res-Net 152 architecture	The first dataset has 170 images the second dataset contains 1300 images	Augmentor Python library for augmentation.	AUC (melanoma: 96, BCC: 91)
[40]	Melanoma/nonmelanoma	SVM-trained, with CNN, extracted features	DermIS dataset and DermQuest data	A median filter for noise removal and CNN for feature extraction	Accuracy (93.75)
[41]	Malignant melanoma/nevus/SK	CNN as single neural-net architecture	ISIC 2017 dataset	CNN ensemble of AlexNet, VGGNet, and GoogleNetfor classification	Average AUC:9 84.8), average accuracy (83.8)
[42]	BCC/nonBCC	CNN	40 FF-OCT images	Trained CNN, consisted of 10 layers for features extraction	Accuracy (95.93), sensitivity (95.2), specificity (96.54)
[43]	Cancerous/noncancerous	CNN	1730 skin lesion and background images	Focused on edge detection	Accuracy (86.67)
[37]	Benign/melanoma	VGG-16 and CNN	ISIC dataset	Dataset was trained on three separate learning models	Accuracy (78)
[44]	Benign/malignant	CNN	ISIC database	ABCD symptomatic checklist for feature extraction	Accuracy (89.5)
[45]	Melanoma/benign keratosis/ melanocytic nevi/BCC/AK/IC/atypical nevi/dermatofibroma/vascular lesions	Deep CNN architecture (DenseNet 201, Inception v3, ResNet 152 and InceptionResNet v2)	HAM10000 and PH2 dataset	Deep learning models outperformed highly trained dermatologists in overall mean results by at least 11%	ROC AUC (DenseNet 201: 98.79–98.16, Inception v3: 98.60–97.80, ResNet 152: 98.61–98.04, InceptionResNet v2: 98.20–96.10)
[46]	Lipoma/fibroma/sclerosis/melanoma	Deep region-based CNN and fuzzy C means clustering	ISIC dataset	Combination of the region-based CNN and fuzzy C-means ensured more accuracy in disease detection	Accuracy (94.8) sensitivity (97.81) specificity (94.17) F1_score (95.89)
[47]	Malignant/benign	6-layers deep CNN	MED-NODE and ISIC datasets	Illumination factor in images affected performance of the system	Accuracy (77.50)
[48]	Melanoma/non melanoma	Hybrid of fully CNN with autoencoder and decoder and RNN	ISIC dataset	Proposed model outperformed state-of-art SegNet, FCN, and ExB architecture	Accuracy (98) Jaccard index (93), sensitivity (95), specificity (94)
[49]	Benign/malignant	2-layer CNN with a novel regularizer	ISIC dataset	Proposed regularization technique controlled complexity by adding a penalty on the dispersion value of classifier’s weight matrix	Accuracy (97.49) AUC (98), sensitivity (94.3), specificity (93.6)
[34]	Malignant melanoma/SK	SVM classification with features extracted with pretrained deep models named AlexNet, ResNet-18, and VGG16	ISIC dataset	SVM scores were mapped to probabilities with logistic regression function for evaluation	Average AUC (90.69)
[12]	Melanoma/BCC/melanocytic nevus/Bowen’s disease/AK/benign keratosis/vascular lesion/dermatofibroma	InceptionResNetV2, PNASNet-5-Large, InceptionV4, and SENet154	ISIC dataset	A trained image-net model was used to initialize network parameters and fine-tuning	Validation Score (76)
[50]	melanoma/BCC/melanocytic nevus/AK/benign keratosis/vascular lesion/dermatofibroma	CNN model with LeNet approach	ISIC dataset	The adaptive piecewise linear activation function was used to increase system performance	Accuracy (95.86)
[51]	Benign/malignant	Deep CNN	ISIC dataset	Data augmentation was performed for data balancing	Accuracy (80.3), precision (81), AUC (69)
[52]	Compound nevus/malignant melanoma	CNN	AtlasDerm, Derma, Dermnet, Danderm, DermIS and DermQuest datasets	BVLC-AlexNet model, pretrained from ImageNet dataset was used for fine-tuning	Mean average precision (70)
[33]	Melanoma/SK	Deep multi-scale CNN	ISIC dataset	The proposed model used Inception-v3 model, which was trained on the ImageNet.	Accuracy (90.3), AUC (94.3)
[53]	Benign/malignant	CNN with 5-fold cross-validation	1760 dermoscopic images	Images were preprocessed on the basis of melanoma cytological findings	Accuracy (84.7), sensitivity (80.9), specificity (88.1)
[32]	Benign/malignant	A very deep residual CNN and FCRN	ISIC 2016 database	FCRN incorporated with a multi-scale contextual information integration technique was proposed for accurate lesions segmentation	Accuracy (94.9), sensitivity (91.1), specificity (95.7), Jaccard index (82.9), dice coefficient (89.7)
[54]	AK/melanocytic nevus/BCC/SK/SCC	CNN	1300 skin lesion images	Mean subtraction for each image, pooled multi-scale feature extraction process and pooling in augmented-feature space	Accuracy (81.8)
[55]	BCC/non-BCC	Pruned ResNet18	297 FF-OCT images	K-fold cross-validation was applied to measure the performance of the proposed system	Accuracy (80)
[56]	Melanoma/non melanoma	ResNet-50 with deep transfer learning	3600 lesion images from the ISIC dataset	The proposed model showed better performance than o InceptionV3, Densenet169, Inception ResNetV2, and Mobilenet	Accuracy (93.5), precision (94) recall (77), F1_ score (85)
[57]	Benign/malignant	Region-based CNN with ResNet152	2742 dermoscopic images from ISIC dataset	Region of interest was extracted by mask and region-based CNN, then ResNet152 is used for classification.	Accuracy (90.4), sensitivity (82), specificity (92.5)

CNN = Convolutional neural network; ISIC = International skin imaging collaboration; SVM = Support vector machine; BCC = Basal cell carcinoma; SCC = Squamous cell carcinoma; AK = Actinic keratosis; IC = Intraepithelial carcinoma; HAM10000 = Human-against-machine dataset with 10,000 images; BVLC = Berkeley Vision and Learning Center; SK= Seborrheic keratosis; FCRN = Fully convolutional residual network; FF-OCT = Full field optical coherence tomography; FCN = Fully convolutional network.

**Table 5:** A comparative analysis of skin cancer detection using KNN-based approaches.

Ref	Skin Cancer Diagnoses	Classifier and Training Algorithm	Dataset	Description	Results (%)
[59]	Melanoma/nevus/normal skin	SOM and feed-forward NN	50 skin lesion images	PCA for decreasing spectra’s dimensionality	Accuracy (96–98)
[60]	BCC, SCC, and melanoma	SOM and RBF	DermQuest and Dermnet datasets	15 features consisting of GCM morphological and color features were extracted	Accuracy (93.15)
[61]	Cancerous/noncancerous	Modified KNN	500 lesion images	Automated Otsu method of thresholding for segmentation	Accuracy (98.3)

SOM = Self organizing map; PCA = Principal component analysis; GCM = Generalized co-occurrence matrices; RBF = Radial Basis Function; KNN = Kohonen self-organizing neural network.

**Table 6:** A comparative analysis of skin cancer detection using GAN-based approaches.

Ref	Skin Cancer Diagnoses	Classifier and Training Algorithm	Dataset	Description	Results (%)
[7]	AK/BCC/benign keratosis/dermatofibroma/melanoma/melanocytic nevus/vascular lesion	GAN	ISIC 2018	The proposed system used deconvolutional network and CNN as generator and discriminator module	Accuracy (86.1)
[8]	Melanoma/nevus/SK	Deep convolutional GAN	ISIC 2017, ISIC 2018, PH^2^	Decoupled deep convolutional GANs for data augmentation	ROC AUC (91.5), accuracy (86.1)
[65]	BCC/vascular/pigmented benign keratosis/pigmented Bowen’s/nevus/dermatofibroma	Self-attention-based PGAN	ISIC 2018	A generative model was enhanced with a stabilization technique	Accuracy (70.1)

GAN = Generative adversarial neural network, PGAN = Progressive generative adversarial network, ROC AUC= Area under the receiver operating characteristic curve.

**Table 7:** Skin Cancer Datasets.

Sr. No	Name of Dataset	Year of Release	No. of Images	Reference Used
1	HAM10000	2018	10,015	[45]
2	PH^2^	2013	200	[45]
3	ISIC archive	2016	25,331	[12,33,34,37,44,46,47,48,49,51,53]
4	DermQuest	1999	22,082	[52,63,67]
5	DermIS		6588	[52,63]
6	AtlasDerm	2000	1024	[52]
7	Dermnet	1998	23,000	[52,59]

## Data Availability

Not applicable as it is a review article, no experiment has been performed by using any data.
